# Supplementary material for: Personalised high tibial osteotomy has mechanical safety equivalent to generic device in a case–control in silico clinical trial
Source: Commun Med (Lond). 2021 Jun 30;1:6. doi: 10.1038/s43856-021-00001-7 (PMC9053187; doi:10.1038/s43856-021-00001-7)
Supplement: Supplementary file 1 — Supplementary Information [file 43856_2021_1_MOESM1_ESM.pdf]

Supplementary Information for:

Personalised high tibial osteotomy has mechanical safety equivalent to generic device in a case-control in silico clinical trial

Alisdair MacLeod[1], Nicholas Peckham[2], Gil Serrancolí[3], Ines Rombach[2], Patrick Hourigan[4], Vipul I Mandalia[4], Andrew D Toms[4], Benjamin J Fregly[5], Harinderjit S Gill*[1,6]

1. Department of Mechanical Engineering, University of Bath, Bath, UK
2. Oxford Clinical Trials Research Unit, NDORMS, University of Oxford, Oxford, UK
3. Department of Mechanical Engineering, Polytechnic University of Catalonia, Barcelona, Catalunya, Spain
4. Royal Devon and Exeter NHS Foundation Trust, Exeter, UK
5. Department of Mechanical Engineering, Rice University, Houston, Texas, USA
6. Centre for Therapeutic Innovation, University of Bath, Bath, UK

*corresponding author email r.gill@bath.ac.uk

Supplementary Figures

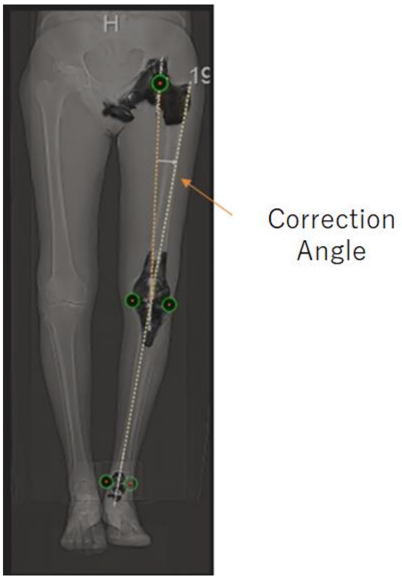

Figure S1: Correction angle assessment using five alignment landmarks from CT data

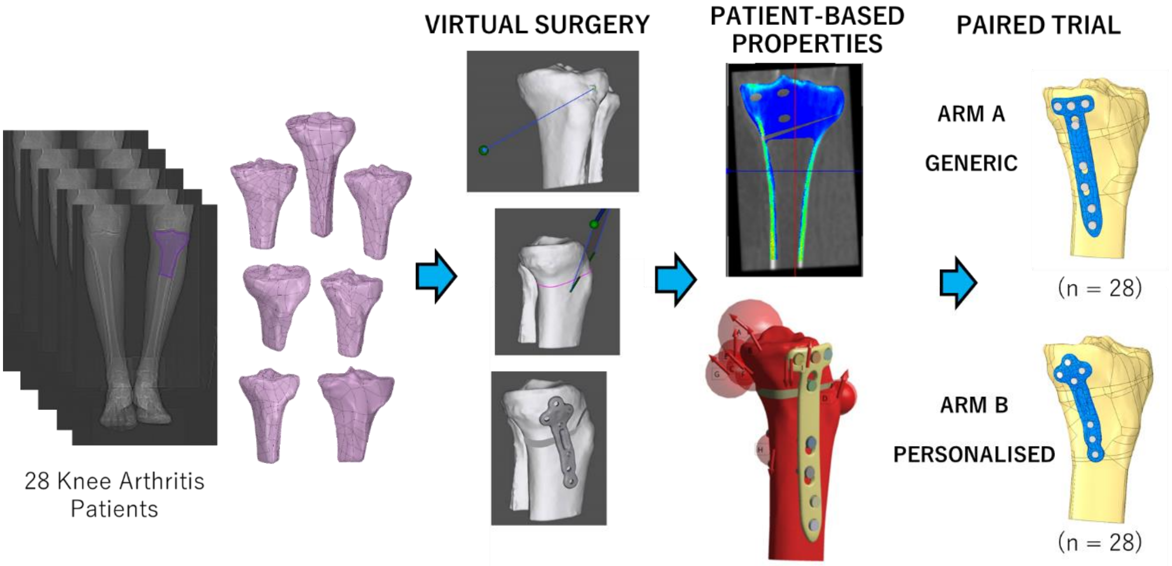

Figure S2: Workflow to generate each of the patient models in the trial

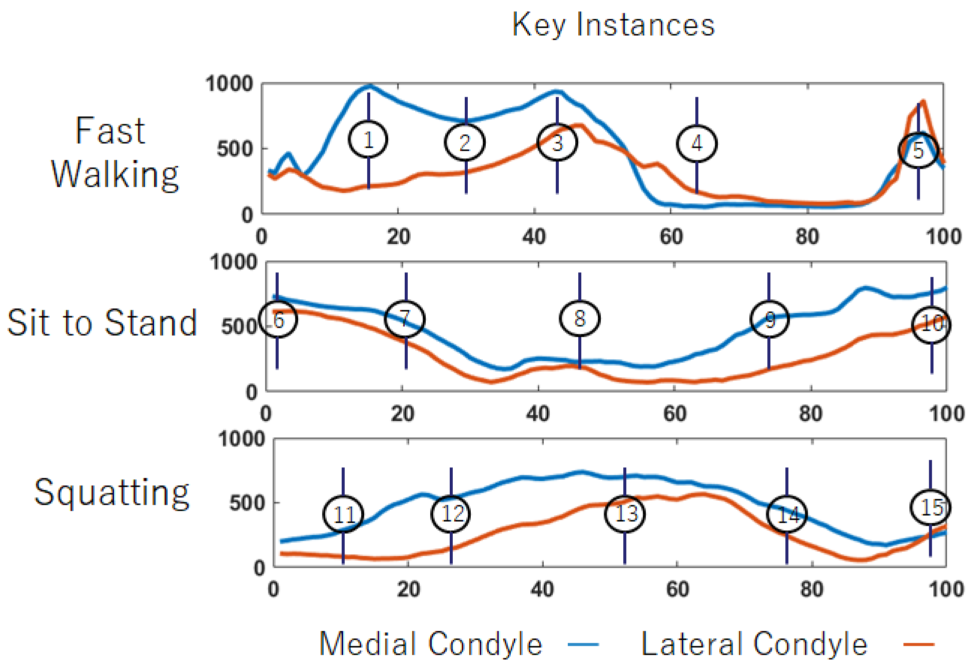

Figure S3: Key instances during the different activities examined in the study. The joint reaction forces (N) acting on the medial and lateral condyles are plotted (y-axis) as a function of the activity cycle time expressed as a percentage (x-axis) for an example patient. For each activity, the five key instances were implemented as load steps applied to each finite element model, the load steps numbers are given in the circles on each activity.

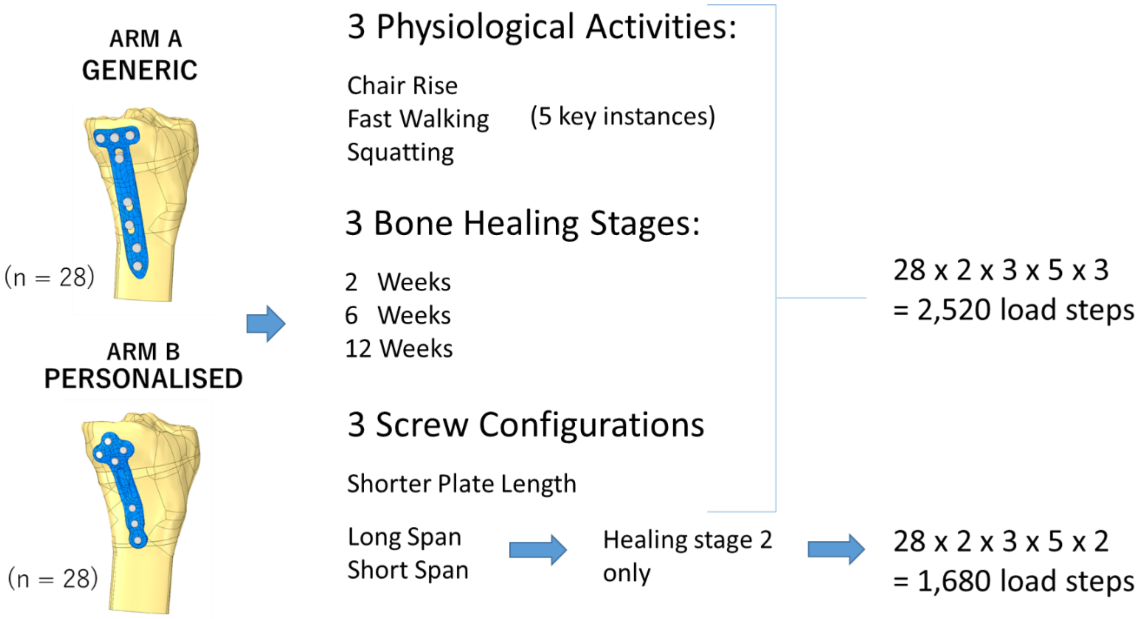

Figure S4: Conditions simulated were three activities, with three different screw configurations, and then three healing stages for screw configuration 3.

Supplementary Tables

Table S1: Demographics of patients in the study, note the correction angle is the virtual correction angle

PATIENT NUMBER	HEIGHT (CM)	WEIGHT (KG)	M/F	AGE (YEARS)	CORRECTION ANGLE (DEGREES)
1	174	77.4	M	72	9.38
2	159	78	F	77	7.50
3	149	70.1	F	84	2.91
4	161	70	F	57	2.03
5	158	75.9	F	69	1.90
6	174	102.2	F	67	2.17
7	161	82.2	F	87	3.66
8	182	103.9	M	69	9.43
9	178	95.1	M	77	6.33
10	191	121.4	M	59	6.38
11	189	108	M	59	5.42
12	173	72.8	M	70	7.96
13	179	105.9	M	66	9.26
14	169	70.8	M	83	13.09
15	173	109.5	M	55	4.70
16	163	81.6	F	71	7.10
17	154	81.1	F	66	2.47
18	184	116	M	64	9.01
19	175	102.4	F	65	8.68
20	160	103.5	F	66	3.86
21	159	70.6	F	62	1.97
22	156	82.5	F	69	4.60
23	164	96.8	F	63	5.41
24	151	86.8	F	53	2.60
25	147	68.8	F	69	9.74
26	184	104.3	M	70	5.26
27	181	110.3	M	50	14.39
28	174	76.1	M	76	0.57
AVERAGE	169	90.1	13 / 28	68	5.99

Table S2: - Material Properties used for the Osteotomy Gap Healing, E=Young's Modulus, v=Poisson's ratio

Hemert Grade (van Hemert et al., 2004)	0		1		2		3		4		5	
Description	Inflamation & Haematoma		Soft Callus		Soft and Hard Callus		Hard Callus Remodelling (healed osteotomy)		Consolidated Callus Remodelling (osteotomy recognisable)		Remodelling (no sign of osteotomy)	
Assumed Material Characteristic	Granulation Tissue		Fibrous Tissue		Cartilage		Immature Bone		Mature Bone		Cortical Bone	
Material Stiffness Values (MPa)	lower	upper	lower	upper	lower	upper	lower	upper	lower	upper	lower	upper
Isaksson et al., 2006	1		2		10		1000		6,000		15,750	
Steiner et al., 2013	0.99	N/A	N/A	3	3.1	200	201	N/A	N/A	8,300	10,000	20,400
Poisson's Ratio, v	0.167	N/A	N/A	0.4	0.167	0.47	0.23	N/A	N/A	0.32	0.325	0.39
Approximate Post Operative Time (van Hemert et al., 2004, Seo et al, 2005)	-		2 weeks		6 weeks		12 weeks		-		-	
Hemert Grade Found at Time Point (Seo 2005)	-		N/A		1.83 ± 0.41		2.67 ± 0.52		-		-	
In Silico Trial Healing Stage	N/A		2		3		4		-		-	
Young's Modulus, E, value adopted (MPa)	-		1.4		24		528		N/A		Bone Mat	
Poisson's Ratio, v, value adopted	-		0.33		0.33		0.3		N/A		0.26	

Table S3 – Joint reaction forces for the different activities included in the study

		MEDIAL CONDYLE FORCE (%BW)			LATERAL CONDYLE FORCE (%BW)			
LOAD STEP	Instance of Activity Cycle (%)	x	y	z	x	y	z	Total Joint Force (% BW)
1	15	18.7	16.6	-150.5	4.8	2.7	-33.2	186.2
2	28	13.9	10.2	-110.2	6.3	3.8	-48.4	160.5
3	44	11.5	5.9	-137.1	18.9	12.2	-100.8	241.0
4	63	0.9	-1.0	-9.6	3.7	-0.4	-26.2	36.1
5	96	-8.9	-17.6	-101.1	38.8	7.1	-130.9	239.8
6	0	15.2	-45.2	-117.3	13.4	-42.5	-99.0	235.1
7	21	9.4	-13.7	-78.5	7.8	-3.1	-54.1	135.0
8	47	4.0	2.2	-36.0	3.7	2.6	-24.4	61.1
9	76	11.3	-7.2	-91.4	4.7	-0.7	-32.2	124.9
10	100	16.8	-49.9	-129.7	12.9	-40.9	-94.9	244.1
11	10	5.5	3.1	-46.5	2.0	1.4	-12.3	59.4
12	26	10.5	-2.4	-84.8	3.5	0.1	-23.7	109.5
13	54	14.5	-45.8	-112.7	11.9	-37.9	-86.3	217.5
14	77	7.7	-1.5	-63.4	4.5	0.3	-31.5	95.6
15	98	4.3	2.2	-40.3	6.8	4.6	-46.3	87.6
