# Supplementary material for: Personalised high tibial osteotomy has mechanical safety equivalent to generic device in a case–control in silico clinical trial
Source: Commun Med (Lond). 2021 Jun 30;1:6. doi: 10.1038/s43856-021-00001-7 (PMC9053187; doi:10.1038/s43856-021-00001-7)
Supplement: Supplementary file 2 — Description of Additional Supplementary Files [file 43856_2021_1_MOESM2_ESM.pdf]

Description of Additional Supplementary Files

File Name: Supplementary Data 1

Description: This zip file contains the data files for the key results. HS2_SC1_2_3_results.txt contains average Von Mises stress for Healing Stage 2 (HS2) for screw configurations (SC) 1, 2 and 3. SC3_HS2_3_4_results.txt contains average Von Mises stress, strain and fracture gap closing for screw configuration 3 (SC3) for healing stages (HS) 2, 3 and 4. The csv files GENERIC_HSX_VMS give the Von Mises Stress for all subjects (columns) for all loading steps (rows) for healing stage X (X=1,2,3), similarly GENERIC_HSX_S1 contains first principle stress (S1) and GENERIC_HSX_S3 contains third principle stress for the generic arm. The equivalent Personalised arm data are in the PERSONALISED_ csv files.
