# Supplementary material for: Personalised high tibial osteotomy has mechanical safety equivalent to generic device in a case–control in silico clinical trial
Source: Commun Med (Lond). 2021 Jun 30;1:6. doi: 10.1038/s43856-021-00001-7 (PMC9053187; doi:10.1038/s43856-021-00001-7)
Supplement: Supplementary file 4 — Reporting Summary [file 43856_2021_1_MOESM4_ESM.pdf]

nature research

Corresponding author(s): Harinderjit Singh Gill

Last updated by author(s): Apr 23, 2021

Reporting Summary

Nature Research wishes to improve the reproducibility of the work that we publish. This form provides structure for consistency and transparency in reporting. For further information on Nature Research policies, see our Editorial Policies and the Editorial Policy Checklist.

Statistics

For all statistical analyses, confirm that the following items are present in the figure legend, table legend, main text, or Methods section.

n/a	Confirmed	
☐	☒	The exact sample size (n) for each experimental group/condition, given as a discrete number and unit of measurement
☐	☒	A statement on whether measurements were taken from distinct samples or whether the same sample was measured repeatedly
☐	☒	The statistical test(s) used AND whether they are one- or two-sided<br>*Only common tests should be described solely by name; describe more complex techniques in the Methods section.*
☐	☒	A description of all covariates tested
☐	☒	A description of any assumptions or corrections, such as tests of normality and adjustment for multiple comparisons
☐	☒	A full description of the statistical parameters including central tendency (e.g. means) or other basic estimates (e.g. regression coefficient) AND variation (e.g. standard deviation) or associated estimates of uncertainty (e.g. confidence intervals)
☒	☐	For null hypothesis testing, the test statistic (e.g. F, t, r) with confidence intervals, effect sizes, degrees of freedom and P value noted<br>*Give P values as exact values whenever suitable.*
☒	☐	For Bayesian analysis, information on the choice of priors and Markov chain Monte Carlo settings
☐	☒	For hierarchical and complex designs, identification of the appropriate level for tests and full reporting of outcomes
☒	☐	Estimates of effect sizes (e.g. Cohen's d, Pearson's r), indicating how they were calculated

Our web collection on statistics for biologists contains articles on many of the points above.

Software and code

Policy information about availability of computer code

Data collection	Segmentation of CT scans - ScanIP M-2017.06, Synopsys Inc., CA, USA; Geometric modelling & virtual surgery - ANSYS SpaceClaim R18.2, ANSYS Inc., PA, USA; Personalised plate creation - HTO Prototype v1.1.11, Renishaw plc, Wotton-under-Edge, Gloucestershire, UK; FE Modelling - ANSYS R18.2, ANSYS Inc., PA, USA; Data extraction - ANSYS APLD R18.2, ANSYS Inc.,PA, USA and Matlab R2017b, The Mathworks, Natick, MA, USA
Data analysis	Matlab R2017b, The Mathworks, Natick, MA, USA; StataCorp. 2019. Stata Statistical Software: Release 15. College Station, TX: StataCorp LLC

For manuscripts utilizing custom algorithms or software that are central to the research but not yet described in published literature, software must be made available to editors and reviewers. We strongly encourage code deposition in a community repository (e.g. GitHub). See the Nature Research guidelines for submitting code & software for further information.

Data

Policy information about availability of data

All manuscripts must include a data availability statement. This statement should provide the following information, where applicable:

- Accession codes, unique identifiers, or web links for publicly available datasets
- A list of figures that have associated raw data
- A description of any restrictions on data availability

Source data for Figures 1, 2, 3 and S3 are provided as Supplementary Data 1. The datasets generated during and/or analysed during the current study (together with muscle loading and code for transforming loads as well as the finite element model from MacLeod et al, ref. a) are available in the University of Bath Research Data Archive, https://doi.org/10.15125/BATH-00926 (ref. b).

a. MacLeod, A.R., Serrancoli, G., Fregly, B.J., Toms, A.D. & Gill, H.S. The effect of plate design, bridging span, and fracture healing on the performance of high tibial

osteotomy plates: An experimental and finite element study. Bone Joint Res 7, 639-649 (2018).

b. Gill, R., MacLeod, A. & Toms, A. Dataset for "Personalised HTO with patient specific plates has mechanical safety equivalent to generic HTO: findings from a novel case-control in silico clinical trial". DOI: https://doi.org/10.15125/BATH-00926. (University of Bath Research Data Archive, Bath, 2021).

Field-specific reporting

Please select the one below that is the best fit for your research. If you are not sure, read the appropriate sections before making your selection.

☒ Life sciences ☐ Behavioural & social sciences ☐ Ecological, evolutionary & environmental sciences

For a reference copy of the document with all sections, see nature.com/documents/nr-reporting-summary-flat.pdf

Life sciences study design

All studies must disclose on these points even when the disclosure is negative.

Sample size	A power analysis was performed using the experimentally measured variation in stiffness and strength for standard sized TomoFix HTO plates ; the TomoFix HTO plate (DePuy Synthes, IN, USA) is a widely implanted HTO device and was used as the generic HTO device in this study. A previous experimental study measuring stiffness of TomoFix bone-plate constructs found the mean and standard deviation to be 1950 N/mm and 577 N/mm respectively. Based on these values and the method of Altman, 25 patients per arm would be needed to give the study 80% power for a detectable difference of 20% in stiffness.
Data exclusions	2 CT scans out of 30 were not sufficient quality and thus lead to loss of two virtual patients
Replication	This is a simulation study, replication is not applicable
Randomization	This was a case-control in silico trial, with the virtual cohort duplicated and each virtual subject acting as their own control
Blinding	This was a case-control in silico trial, so blinding was not possible

Reporting for specific materials, systems and methods

We require information from authors about some types of materials, experimental systems and methods used in many studies. Here, indicate whether each material, system or method listed is relevant to your study. If you are not sure if a list item applies to your research, read the appropriate section before selecting a response.

Materials & experimental systems

n/a	Involved in the study	
☒	☐	Antibodies
☒	☐	Eukaryotic cell lines
☒	☐	Palaeontology and archaeology
☒	☐	Animals and other organisms
☐	☒	Human research participants
☐	☒	Clinical data
☒	☐	Dual use research of concern

Methods

n/a	Involved in the study	
☒	☐	ChIP-seq
☒	☐	Flow cytometry
☒	☐	MRI-based neuroimaging

Human research participants

Policy information about studies involving human research participants

Population characteristics	Patient data (CT scans and demographics) were obtained from patients with radiologically confirmed moderate to severe knee osteoarthritis presenting at a specialist orthopaedic centre (Princess Elizabeth Orthopaedic Centre, Royal Devon and Exeter NHS Foundation Trust, Exeter, UK). Patients were 50 to 87 years old (mean: 68), 54% female, 68.8 to 121.4 kg (mean: 90.1 kg), 147 to 190 cm tall (mean: 169 cm) and had no history of knee surgery
Recruitment	The basis of ethical approval was based on anonymous re-use of existing data, PACS database was searched for patients that met the inclusion criteria and had given consent for anonymous data re-use.
Ethics oversight	HRA - REC reference: 17/HRA/0033, RD&E NHS, UK

Note that full information on the approval of the study protocol must also be provided in the manuscript.

Clinical data

Policy information about clinical studies

All manuscripts should comply with the ICMJE guidelines for publication of clinical research and a completed CONSORT checklist must be included with all submissions.

Clinical trial registration	ClinicalTrials.gov NCT03419598
Study protocol	Available from ClinicalTrials.gov NCT03419598
Data collection	Imaging data collected at Royal Devon and Exeter NHS Trust, modelling, simulation and data reduction performed at the University of Bath, UK.
Outcomes	Since investigating mechanical safety was the purpose, mechanical stress in the high tibial osteotomy plates was chosen as the primary outcome variable as exceeding the fatigue limit will increase risk of failure, with secondary outcome variables of strain in the bone adjacent to the screws which is indicative of fixation failure, and micro-motion at the fracture gap which is indicative of mechanical stability.
